# Impact of the COVID-19 pandemic on vancomycin-resistant *Enterococcus* bloodstream infections: a 6-year study in Western Greece

**DOI:** 10.3389/fmicb.2025.1656334

**Published:** 2025-10-24

**Authors:** Maria Lagadinou, Christos Michailides, Christodoulos Chatzigrigoriadis, Ioannis Erginousakis, Prodromos Avramidis, Marina Amerali, Fotini Tasouli, Anna Chondroleou, Katerina Skintzi, Anastasia Spiliopoulou, Fevronia Kolonitsiou, Leonidia Leonidou, Stelios F. Assimakopoulos, Markos Marangos

**Affiliations:** ^1^Department of Internal Medicine, University of Patras, Patras, Greece; ^2^Medical School of Patras, University of Patras, Patras, Greece; ^3^Nurse Infection Control, University of Patras, Patras, Greece; ^4^Department of Microbiology, University of Patras, Patras, Greece; ^5^Division of Infectious Diseases, University of Patras, Patras, Greece

**Keywords:** antimicrobial resistance, gram positive, antibiotics, bacteremia, *Enterococcus* spp.

## Abstract

**Background:**

Antimicrobial resistance is a critical and growing global health concern. While drug-resistant Gram-negative bacilli pose a significant threat, multidrug-resistant Gram-positive bacteria—such as methicillin-resistant *Staphylococcus aureus* (MRSA), vancomycin-resistant *Enterococcus* (VRE), and β-lactamase-resistant *Streptococcus pneumoniae*—also present serious clinical challenges.

**Aim:**

This study provides an epidemiological analysis of resistant Gram-positive bacteria, focusing on VRE, at a tertiary university hospital in Western Greece from 2018 to 2023.

**Results:**

A total of 276 blood cultures with vancomycin-resistant *Enterococcus* spp. were recorded. A significant increase in VRE prevalence was observed in intensive care units (ICUs), with cases rising from 4 in 2020 to 36 in 2021. A broader increase across medical and surgical wards was noted in 2022–2023. Linezolid resistance remained low throughout the study period. Mortality data revealed a marked increase in deaths after 2020 compared to 2018–2019, coinciding with the rise in VRE-related bloodstream infections. The Coronavirus Disease 2019 (COVID-19) pandemic was associated with higher VRE rates in ICU patients, likely due to prolonged hospitalizations, increased use of invasive devices, and broad-spectrum antibiotic use. Resistance rates to both linezolid and tigecycline remained low, while daptomycin resistance showed an increasing trend the same period.

**Conclusion:**

The number of VRE increased over the study period. Linezolid and tigecycline remained largely effective, but emerging resistance patterns—particularly to daptomycin—underscore the urgent need for strengthened antimicrobial stewardship and the development of novel therapeutic options to address rising resistance among Gram-positive pathogens.

## 1 Introduction

Antimicrobial resistance (AMR) represents a critical global health threat, causing up to 10 million deaths annually by 2050 (Amberpet et al., 2019). While drug-resistant Gram-negative bacilli are a well-recognized concern, multidrug-resistant Gram-positive organisms—such as methicillin-resistant *Staphylococcus aureus* (MRSA), vancomycin-resistant *Enterococcus faecium* (VRE), and β-lactamase-resistant *Streptococcus pneumoniae*—also pose serious clinical challenges (Zhang et al., 2023a).

Gram-positive bacteria are particularly problematic due to their high genetic adaptability, enabling them to acquire resistance to nearly all available antimicrobial agents. This makes resistance among these pathogens a persistent threat that necessitates ongoing surveillance to identify emerging resistance mechanisms and inform rational antibiotic use. Of particular concern in addition to methicillin-resistant *Staphylococcus aureus* (MRSA) and vancomycin-resistant *Staphylococcus aureus* (VRSA) is vancomycin-resistant *Enterococcus* (VRE) (Zhang et al., 2023a).

VRE have emerged as a significant global nosocomial threat, nearly three decades after their initial identification in Europe in the late 1980s (Al Rubaye et al., 2021). To date, eight distinct acquired vancomycin resistance gene clusters have been identified—vanA, vanB, vanD, vanG, vanE, vanL, vanM, and vanN (Al Rubaye et al., 2021). In contrast, the vanC gene cluster is intrinsic to *Enterococcus casseliflavus* and *Enterococcus gallinarum* which exhibit low-level resistance to vancomycin (MIC 8–32 mg/L) while remaining susceptible to teicoplanin.

Generally, van gene clusters encode three groups of functionally related enzymes: (1) enzymes involved in the synthesis of altered peptidoglycan precursors, (2) enzymes that eliminate the native D-Ala-D-Ala termini, and (3) a two-component regulatory system that enables inducible resistance (Al Rubaye et al., 2021). Specifically, *Enterococcus* species exhibiting high-level resistance to vancomycin (MIC ≥ 64 mg/L) and to teicoplanin (MIC ≥ 16 mg/L) are classified under the VanA phenotype. Species with variable resistance to vancomycin (MIC 4–64 mg/L) but remaining susceptible to teicoplanin are classified as VanB phenotype. It is now known that levels of vancomycin resistance among VanB isolates may range from 4 to > 1,000 μg/ml while susceptibility to teicoplanin resistance is retained. The VanC resistance phenotype was described in *E. casseliflavus* and *E. gallinarum* which demonstrate intrinsic, low-level resistance to vancomycin (MIC 4 to 32 μg/ml) and are susceptible to teicoplanin. The VanD phenotype is characterized by intrinsic high-level resistance to both vancomycin and teicoplanin (Al Rubaye et al., 2021; Cetinkaya et al., 2000).

An alarming synergy has been observed between SARS-CoV-2 infection and *Enterococcus* species. Evidence suggests that SARS-CoV-2 pneumonia may disrupt the gut microbiota, promote the overgrowth of *Enterococcus* spp. and increase intestinal permeability. These alterations may help explain the observed increase in bloodstream infections (BSIs) caused by *Enterococcus* spp. in patients with COVID-19 (Toc et al., 2022). Furthermore, the COVID-19 pandemic has disrupted antimicrobial stewardship and infection prevention programs globally, raising concerns about the acceleration of AMR. An unexpected consequence of the pandemic has been the increased incidence of *Enterococcus* infections in patients hospitalized with COVID-19 (Toc et al., 2022).

In this study, we aimed to conduct a comprehensive epidemiological analysis of VRE bloodstream infections at a tertiary university hospital in Western Greece, focusing on the period before and after the onset of the COVID-19 pandemic (2018–2023). Our objectives included evaluating the distribution of VRE across hospital departments and analyzing their antimicrobial susceptibility patterns to key therapeutic agents (Amberpet et al., 2019; Reffat et al., 2024).

## 2 Materials and methods

### 2.1 Study design and population

This retrospective observational study analyzed all bloodstream isolates of VRE obtained from patients hospitalized at the University General Hospital of Patras (a tertiary care center in Western Greece) between January 2018 and December 2023. A total of 276 blood cultures with VRE were recorded. The patient search was conducted using a specialized database from the Infection Control Nursing Department, which records all multidrug-resistant pathogens in our hospital (pathogen type, resistance to representative antibiotics, infection site). For the purposes of this study, we selected bloodstream infections caused by VRE species.

Patient records were obtained from four key departments: Medical Wards (MW): Including Internal Medicine, Cardiology, Nephrology, Neurology, Hematology-Oncology, and the Hematopoietic Stem Cell Transplantation Unit, Surgical Departments (SD): Covering General Surgery, Orthopedics, Obstetrics, Neurosurgery, and Urology, the adult Intensive Care Unit (ICU), the Neonatal Intensive Care Unit (NICU), and Pediatric Intensive Care Unit (PICU). The search for antibiotic susceptibility profiles, for the recording of resistance, was conducted through the hospital's central information system for each patient individually.

Identification of *Enterococcus* spp. was performed by VITEK^®^ 2 Gram-positive identification cards (bioMérieux, Marcy-l' Etoile, France), an automated system for the identification of microorganisms and antimicrobial susceptibility testing (AST). Antimicrobial susceptibility testing regarding daptomycin was also performed using the concentration gradient diffusion assay (E-test bioMérieux SA, France). According to the European Committee on Antimicrobial Susceptibility Testing (EUCAST), VRE were defined as *Enterococcus* spp. with a vancomycin Minimal Inhibitory Concentration (MIC) ≥ 4 mg/L. Tigecycline resistance to *Enterococcus* spp. was considered a MIC > 0.25 mg/L.

The study was conducted in accordance with the Hospital Research Ethics Committee's guidelines (Approval Number PN: 10408/10.07.2023), the Declaration of Helsinki, and STROBE guidelines.

### 2.2 Statistical analyses

Data were statistically analyzed using the Statistical Package for the Social Sciences (SPSS), version 22.0 for Windows (IBM Corp., Armonk, NY, USA). Data were presented as mean ± standard deviation (SD). A *p*-value of less than 0.05 was considered statistically significant. Frequencies and percentages of resistant bacteria were determined for all clinical isolates. For the calculation of statistical differences, the chi-square test was used.

## 3 Results

### 3.1 Vancomycin resistant *Enterococcus*

**Figure 1:**
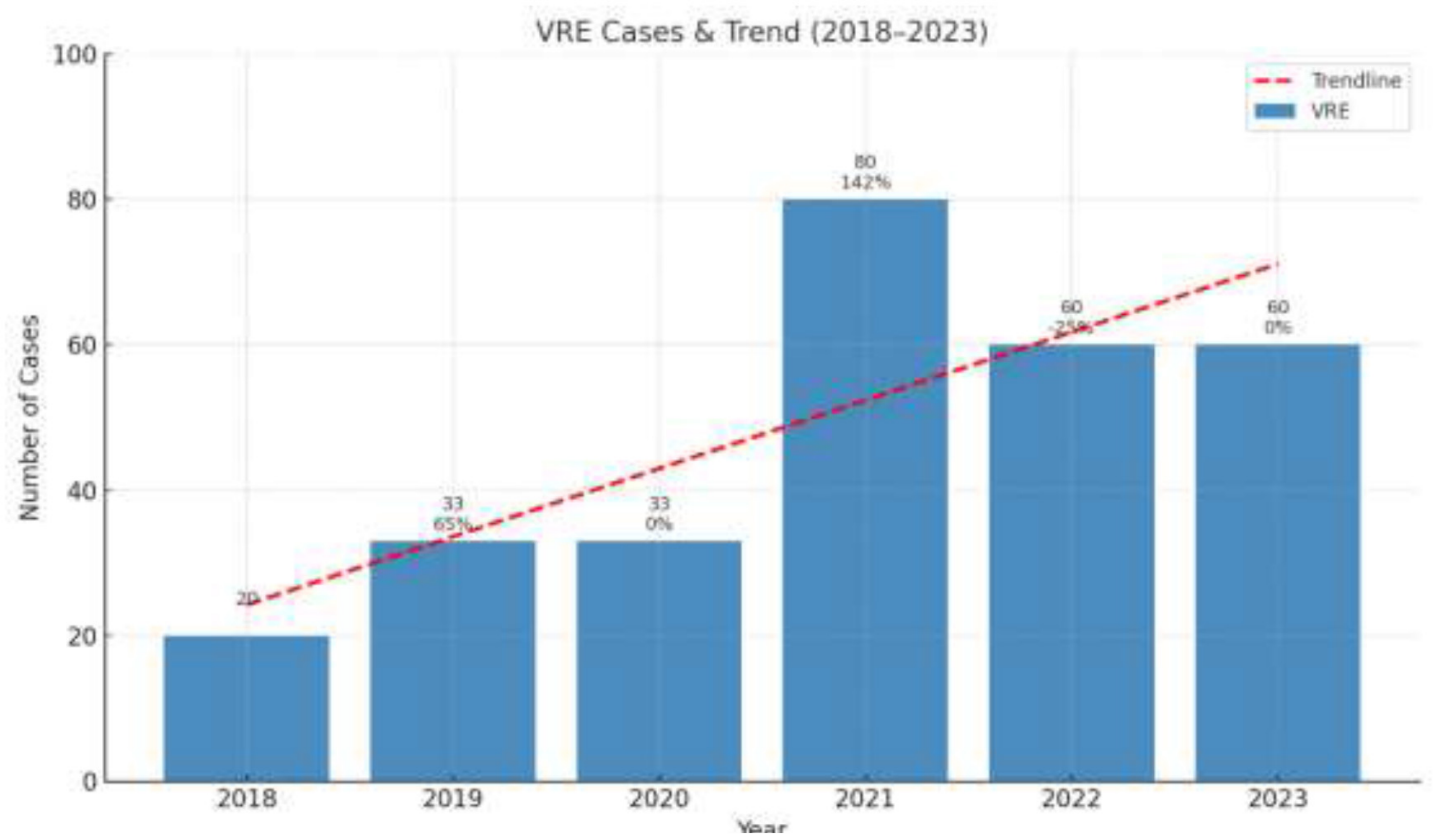
Annual number of cases of Vancomycin Resistant *Enterococcus* per year (pre-COVID and post-COVID period). The values in the Figure represent the absolute number of cases per year.

During the study period, a total of 276 blood cultures with VRE were recorded. The annual number of cases of VRE are shown in Figure 1.

**Figure 2:**
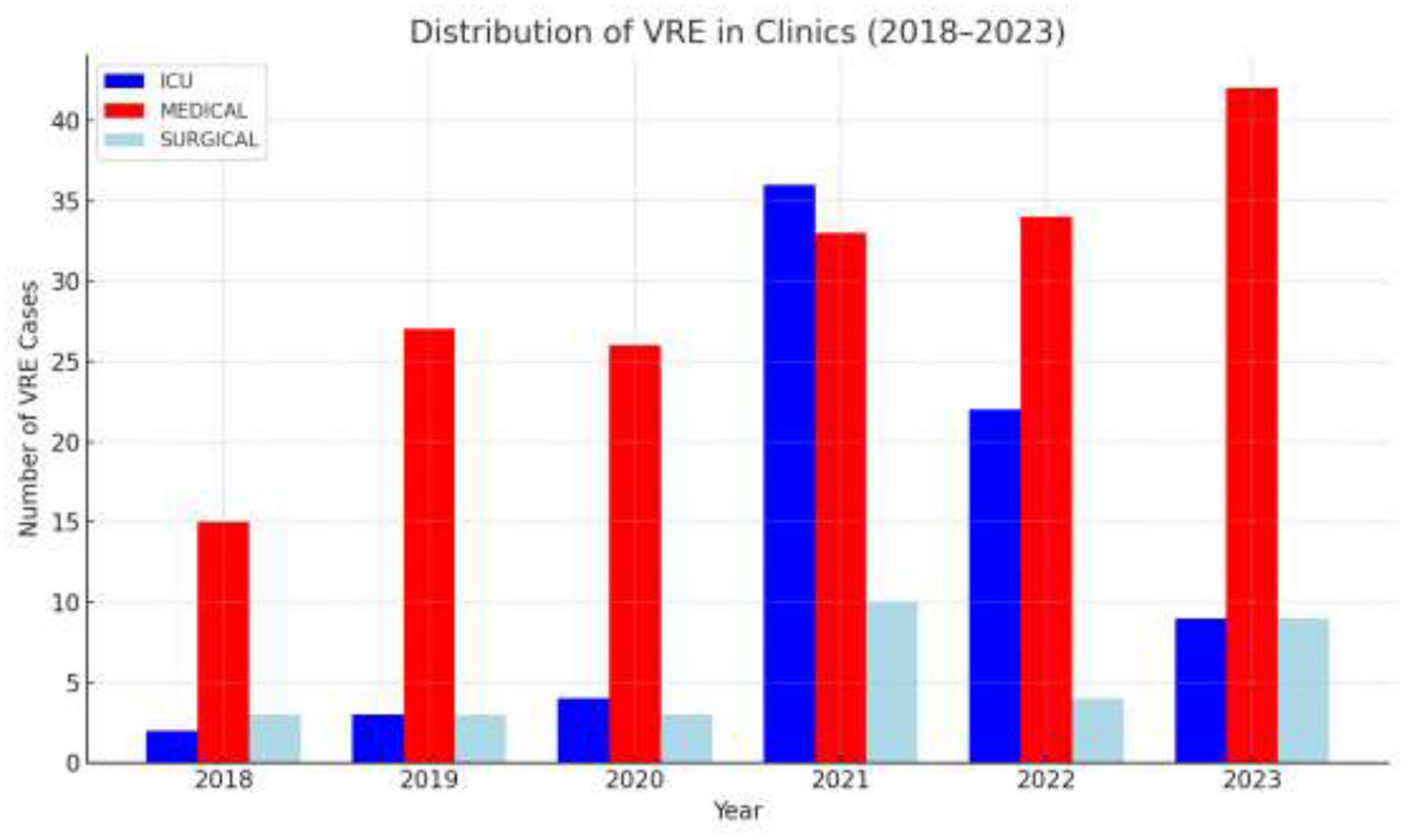
Annual distribution of VRE BSI cases by department and year. ICU: intensive care unit (Neonatal and adults); Medical wards: including Internal Medicine, Cardiology, Nephrology, Neurology, Hematology–Oncology and Hematopoietic Stem Cell Transplantation Unit; Surgical wards: including orthopedics, neurosurgery, general surgery.

**Figure 3:**
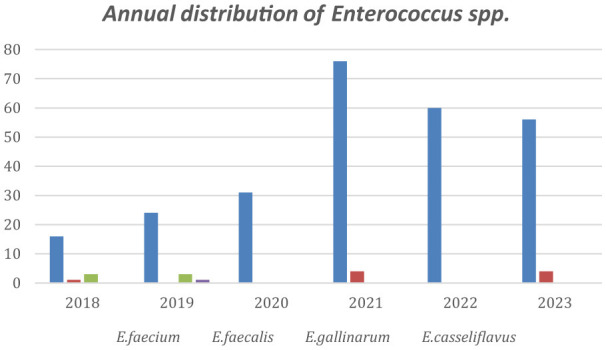
Annual distribution of *Enterococcus* spp. cases.

The annual distribution of VRE in every department is shown in Figure 2 while Figure 3 shows the annual distribution of VRE species. Notably, a substantial increase in VRE cases was observed after the onset of COVID-19 pandemic. A statistically significant rise in VRE bacteremia incidence was recorded in all the examined wards during the post-COVID period, especially in the years 2021, 2022 and 2023 (*p* = 0.04), followed by a remarkable annual decrease in ICUs since 2021. Although an upward trend in VRE cases was also noted in medical and surgical departments during 2021–2023, these increases did not reach statistical significance (*p* **=** 0.881 and *p* = 0.931, respectively). Moreover, as shown in Figure 3, *Enterococcus faecium* the predominant species across all over the study period, with a marked increase after the onset of COVID-19 pandemic.

### 3.2 Mortality associated with VRE BSIs per year

**Figure 4:**
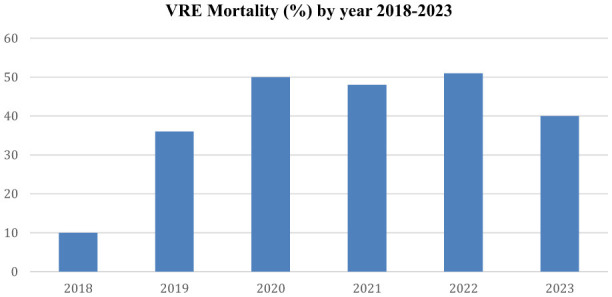
The figure displays the annual mortality of patients with VRE-related bacteremia from 2018 to 2023.

Mortality data collected during the study period revealed a marked increase in deaths occurring after 2020 compared to 2018–2019, which corresponds temporally with the observed rise in VRE-related bloodstream infections during the same period. The annual mortality is shown in Figure 4.

### 3.3 Antimicrobial resistance rates of VRE BSI isolates

**Figure 5:**
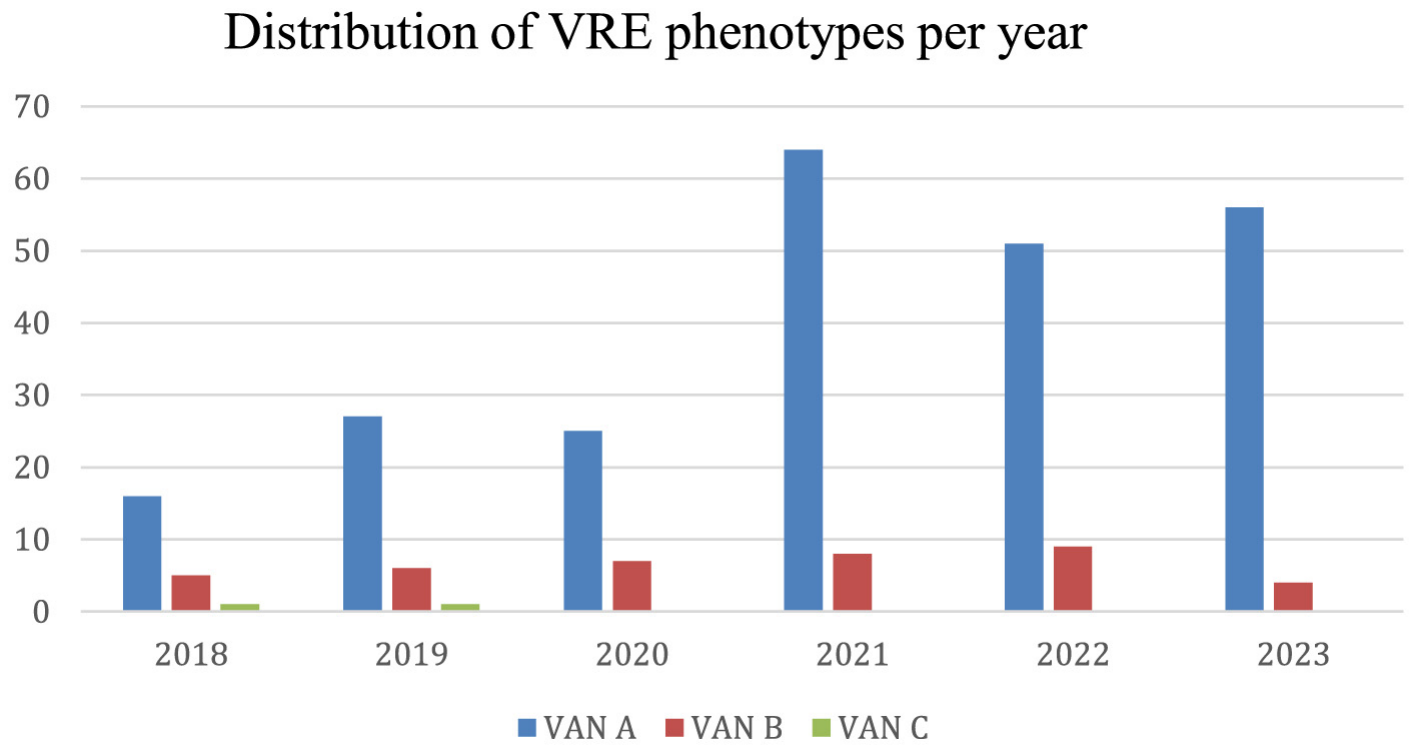
Distribution of VRE phenotypes per year. The distribution refers to the *Enterococcus* exhibiting the VanA, VanB or VanC phenotypes.

**Figure 6:**
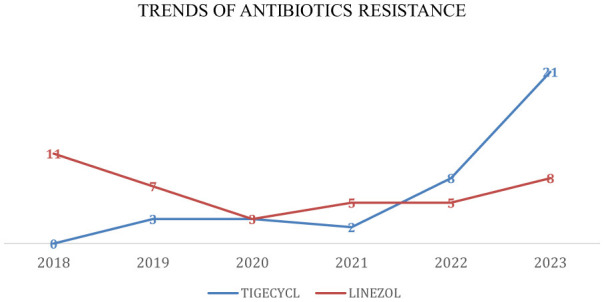
Trends of antibiotic resistance of *Enterococcus* spp., isolated in BSIs during 2018–2023. Tigecycl, Tigecycline; Linezol, Linezolid.

Antibiotic resistance rates for VRE are presented in Table 1 and Figures 5, 6. Resistance to linezolid ranged from 3% to 11%. Teicoplanin resistance rates were notably high, at 83%, 81%, and 85% in 2021, 2022, and 2023, respectively. *Enterococcus* spp. showed very high susceptibility to tigecycline in 2019, 2020, 2021, and 2022 (Table 1, Figure 4). Interestingly, the increase in teicoplanin resistance observed since 2019 was followed by a rise in tigecycline resistance initiating in 2023.

**Table 1:** Antimicrobial resistance (%) of VRE species isolated in BSIs during 2018–2023.

**Type ofantibiotic**	**Year**	**Resistantrate (%)**
Tigecycline	2018	(no data)
	2019	3
	2020	3
	2021	2
	2022	8
	2023	21
Linezolid	2018	11
	2019	7
	2020	3
	2021	5
	2022	5
	2023	8

**Figure 7:**
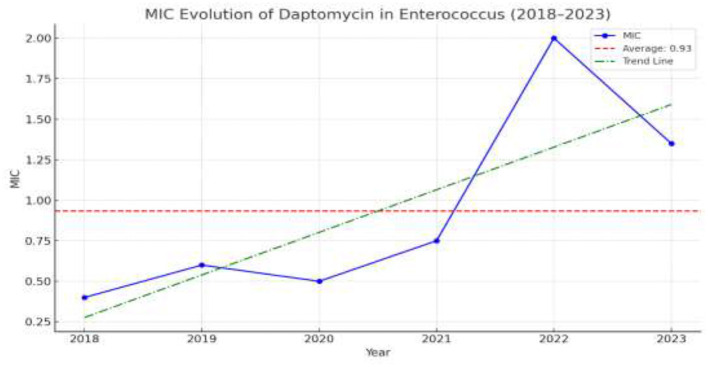
The escalating resistance of VRE in daptomycin. All values represent the median MIC for daptomycin.

MIC trends for daptomycin resistance among VRE species were stably under 0.75 mg/L until 2020, when an uprising initiated peaking in 2022 with a median MIC = 2 mg/L. In 2023, the proportion of *Enterococcus* isolates with daptomycin minimum inhibitory concentrations (MICs) >4 mg/L ranged from 11% to 45%, suggesting a potentially emerging resistance trend, reflecting previous years' increasing resistance, with a hopeful slight decrease in median MIC after 2022. Figure 7 illustrates the rising median MIC values for daptomycin among VRE isolates over the years of study.

## 4 Discussion

This study investigated the detection rates and antimicrobial resistance patterns of VRE at a tertiary university hospital in Western Greece over a 6-year period (2018–2023). Although the COVID-19 pandemic appears to have subsided, its impact on hospital-acquired infections caused by multidrug-resistant pathogens remains a subject of ongoing debate.

*Enterococcus* is an opportunistic pathogen and a well-established cause of nosocomial infections, particularly in critically ill patients. Since VRE remains under constant surveillance by healthcare professionals, several protocols have been proposed to avoid its spread. However, the increased pressure on healthcare systems during the COVID-19 pandemic has been associated with documented breaches in infection control practices, contributing to hospital-wide dissemination of VRE (Toc et al., 2023).

Our findings revealed a progressive increase in the number of VRE isolates across three major hospital departments. The increasing number of VRE infections caused by *Enterococcus* spp., was reported also by Polemis et al. in samples by other hospitals in Greece. The upward trend in vancomycin and teicoplanin resistance starting in 2021 was observed in this study with a 33% increase for both regimens (Polemis et al., 2021). These results are particularly concerning, as the World Health Organization (WHO) has classified resistant Gram-positive organisms as “high priority” pathogens due to their rapid development of resistance and their significant contribution to global morbidity and mortality (Tacconelli et al., 2018).

In our study, we observed a clear upward trend in VRE bloodstream infections (BSIs) after 2020, particularly in intensive care units (ICUs). This trend is consistent with surveillance data from WHONET Greece, which also reported a rise in *Enterococcus* BSIs among ICU patients during the COVID-19 pandemic (Polemis et al., 2021). Similar observations have been documented internationally. Studies of ICU-treated COVID-19 patients reported significantly higher rates of VRE isolation from both mono- and polymicrobial BSIs compared to pre-pandemic levels (20–33%) [Bonazzetti et al., 2021; DeVoe et al., 2022; European Centre for Disease Prevention Control (ECDC), 2018].

Our study identified a significant increase in VRE bacteremia in 2021 and 2022 across all departments, with the most pronounced rise observed in ICUs. A study conducted by Karakosta et al. reported similar findings: in non-ICU settings, VRE BSI rates increased from 0.03 infections per 1,000 patient-days (pd) in 2019 to 0.17 in 2021 and 1.07 in 2022. In ICUs, the rate rose from 0.44 infections/1,000 pd in 2019 to 1.56 in 2021 and 1.03 in 2022, reflecting a substantial increase of approximately 260% in ICUs and 380% in non-ICU settings in 2021 (Karakosta et al., 2024).

The VRE mortality diagram provides useful data regarding the clinical impact of *Enterococcus* spp. infections. Notably, the pandemic was associated with a marked rise in VRE BSI incidence and overall colonization/infection rates in 2021 and 2022, accompanied by increased mortality. These findings are consistent with results from other clinical studies and systematic reviews that also detected this post-COVID-19 era upward trend (Karakosta et al., 2024; Abubakar et al., 2023; Micheli et al., 2023; Grasselli et al., 2021). In a recent meta-analysis, (Eichel et al. 2023) reported significantly higher mortality for VRE *faecium* BSIs compared to vancomycin-susceptible *faecium* (VSE) BSIs (risk ratio [RR] 1.46; 95% confidence interval [CI]: 1.17–1.82), while no significant difference was observed between VRE *faecium* and VRE *faecalis* BSIs (RR 1.00; 95% CI: 0.52–1.93).

A plausible explanation for the observed increase in VRE prevalence during and after the pandemic is the heightened vulnerability of COVID-19 patients, who often require prolonged hospital stays, ICU admission, central venous catheters (especially femoral one), and mechanical ventilation—all of which are known risk factors for bloodstream infections. In addition, the intense pressure on healthcare systems during the pandemic led many clinicians to rely heavily on empirical antibiotic therapy to manage suspected bacterial infections (Langford et al., 2020; Buetti et al., 2021; Weiner-Lastinger et al., 2022).

Recent studies have shown that over 70% of hospitalized COVID-19 patients received antibiotics, most commonly broad-spectrum agents such as fluoroquinolones and third-generation cephalosporins (Karakosta et al., 2024; Langford et al., 2020). This widespread and frequently untargeted use of antimicrobials likely contributed to selective pressure, promoting the emergence and spread of multidrug-resistant organisms, including VRE.

It is also important to underscore the role of gut microbiome dysbiosis in COVID-19 patients, which may help explain the observed rise in bloodstream infections caused by *Enterococcus* spp. SARS-CoV-2 infection causes significant disruptions to the gut microbiota, resulting in dysbiosis, characterized by microbial imbalance (Ancona et al., 2023; Smail et al., 2025). On one hand, recent studies have reported a reduction in beneficial commensal bacteria such as *Faecalibacterium prausnitzii* and *Bifidobacterium* spp., alongside an increase in opportunistic pathogens, including *Enterococcus* and *Escherichia coli*, in patients with COVID-19 (Smail et al., 2025; Zhang et al., 2023b; Wang et al., 2023; Lamers et al., 2020). On the other hand, growing evidence indicates that SARS-CoV-2 can directly infect enterocytes—key regulators of gut microbiota. Damage to enterocytes caused by infection or inflammation can compromise their function, leading to dysbiosis and increased intestinal permeability (“leaky gut”) (Ra and Bang, 2024), thereby promoting the translocation of opportunistic and overgrown pathogens like *Enterococcus* into the systemic circulation.

Given these challenges, linezolid has emerged as a key therapeutic option for managing infections caused by resistant Gram-positive pathogens. Encouragingly, susceptibility to oxazolidinones (linezolid) among *Enterococcus* species has remained consistently high. In our study, linezolid demonstrated high *in vitro* activity, with resistance rates ranging from 3% to 14% for *Enterococcus* spp. These findings support the continued use of linezolid as a reliable agent in settings where vancomycin resistance is prevalent.

The lowest resistance rates were reported for tigecycline. Our findings are in line with those of Toc et al., who observed no tigecycline-resistant *Enterococcus* spp. strains in the pre-COVID period, whereas resistance emerged in the post-COVID periods from 1st May 2021 to 30th October 2021 and from 1st November 2021 to 30th April 2022 (Al Rubaye et al., 2021; Buetti et al., 2022).

Limitations of our study include the retrospective nature of the data analysis. Furthermore, it is important to mention that all the data included are from only one academic hospital in Greece. Additionally, the unavailability of MIC data for new agents limited our ability to categorize microorganisms strictly according to standard definitions. Moreover, our results are based on the evaluation of antimicrobial susceptibility phenotypes and were not correlated to the presence of resistance genes. Finally, no molecular techniques were used to identify the diversity of VRE strains.

In conclusion, the total number of Gram-positive bacteria isolated increased over the 6-year study period with a marked emergence of highly resistant *Enterococcus* strains. This study highlights the steady rise in the emergence of highly resistant *Enterococcus* species. While high efficacy against glycopeptides and linezolid was observed among *Enterococcus* spp. isolates, the gradual increase in resistance to these last-line antibiotics in multidrug-resistant Gram-positive pathogens underscores the urgent need for new antibiotic research to address these challenging infections. As previously highlighted, the unregulated use of antibiotics continues to evolve and plays an increasingly critical role in the emergence of highly resistant bacterial strains. In the context of analyzing the aftermath of the COVID-19 pandemic, it becomes essential to assess the contribution of prior antibiotic use to the development of resistant pathogens capable of causing severe infections (Reffat et al., 2024).

## Data Availability

The datasets presented in this article are available without restrictions. Requests to access the datasets should be directed to: m_lagad2004@yahoo.gr.

## References

[B1] Abubakar U. Al-Anazi M. Alanazi Z. Rodríguez-Baño J. (2023). Impact of COVID-19 pandemic on multidrug-resistant Gram-positive and Gram-negative pathogens: a systematic review. J. Infect. Public Health 16, 320–331. 10.1016/j.jiph.2022.12.022 36657243 PMC9804969

[B2] Al Rubaye M. T. S. Janice J. Bjørnholt J. V. Jakovljev A. Hultström M. E. Sundsfjord A.. (2021). Novel genomic islands and a new vanD subtype in the first sporadic VanD-type vancomycin-resistant enterococci in Norway. PLoS One 16:e0255187. 10.1371/journal.pone.0255187 34297779 PMC8301612

[B3] Amberpet R. Sistla S. Sugumar M. Nagasundaram N. Manoharan M. Parija S. C.. (2019). Detection of heterogeneous vancomycin-intermediate Staphylococcus aureus: a preliminary report from South India. Indian J. Med. Res. 150, 194–198. 10.4103/ijmr.IJMR_1976_17 31670275 PMC6829776

[B4] Ancona G. Alagna L. Alteri C. Palomba E. Tonizzo A. Pastena A.. (2023). Gut and airway microbiota dysbiosis and their role in COVID-19 and long-COVID. Front Immunol. 14:1080043. 10.3389/fimmu.2023.1080043 36969243 PMC10030519

[B5] Bonazzetti C. Morena V. Giacomelli A. Oreni L. Casalini G. Galimberti L. R.. (2021). Unexpectedly high frequency of enterococcal bloodstream infections in coronavirus disease 2019 patients admitted to an Italian ICU: an observational study. Crit Care Med. 49, e31–e40. 10.1097/CCM.0000000000004748 33122577 PMC7737701

[B6] Buetti N. Ruckly S. de Montmollin E. Reignier J. Terzi N.. (2021). COVID-19 increased the risk of ICU-acquired bloodstream infections: a case-cohort study from the multicentric OUTCOMEREA network. Intensive Care Med. 47, 180–187. 10.1007/s00134-021-06346-w 33506379 PMC7839935

[B7] Buetti N. Tabah A. Loiodice A. Ruckly S. Aslan A. T. Montrucchio G. . (2022). Different epidemiology of bloodstream infections in COVID-19 compared to non-COVID-19 critically ill patients: a descriptive analysis of the Eurobact II study. Crit Care. 26:319. 10.1186/s13054-022-04166-y 36258239 PMC9578203

[B8] Cetinkaya Y. Falk P. Mayhall C. G. (2000). Vancomycin-resistant enterococci. Clin Microbiol Rev. 13, 686–707 10.1128/CMR.13.4.686 11023964 PMC88957

[B9] DeVoe C. Segal M. R. Wang L. Stanley K. Madera S.. (2022). Increased rates of secondary bacterial infections, including *Enterococcus* bacteremia, in patients hospitalized with coronavirus disease 2019 (COVID-19). *Infect Control Hosp. Epidemiol*. 43, 1416–1423. 10.1017/ice.2021.391 34486503 PMC8458844

[B10] Eichel V. M. Last K. Bruhwasser C. H. von Baum H. Dettenkofer M. Götting T.. (2023). Epidemiology and outcomes of vancomycin-resistant enterococcus infections: a systematic review and meta-analysis. J. Hosp. Infect. 141, 119–128. 10.1016/j.jhin.2023.09.008 37734679

[B11] European Centre for Disease Prevention and Control (ECDC). (2018). Surveillance of antimicrobial resistance in Europe—Annual report of the European Antimicrobial Resistance Surveillance Network (EARS-Net). Stockholm: ECDC.

[B12] Grasselli G. Scaravilli V. Mangioni D. Scudeller L. Alagna L.. (2021). Hospital-acquired infections in critically ill patients with COVID-19. Chest 160, 454–465. 10.1016/j.chest.2021.04.002 33857475 PMC8056844

[B13] Karakosta P. Vourli S. Kousouli E. Protonotariou E. Tarpatzi A. Vourli S.. (2024). Multidrug-resistant organism bloodstream infection and hospital acquisition among inpatients in three tertiary Greek hospitals during the COVID-19 era. Eur. J. Clin. Microbiol. Infect Dis. 43, 1241–1246. 10.1007/s10096-024-04806-x 38530465 PMC11178613

[B14] Lamers M. M. Beumer J. van der Vaart J. Knoops K. Puschhof J. Breugem T. I.. (2020). SARS-CoV-2 productively infects human gut enterocytes. Science 369, 50–54. 10.1101/2020.04.25.060350 32358202 PMC7199907

[B15] Langford B. J. So M. Raybardhan S. Leung V. Westwood D.. (2020). Bacterial co-infection and secondary infection in patients with COVID-19: a living rapid review and meta-analysis. Clin Microbiol Infect. 26, 1622–1629. 10.1016/j.cmi.2020.07.016 32711058 PMC7832079

[B16] Micheli G. Sangiorgi F. Catania F. Chiuchiarelli M. Frondizi F.. (2023). The hidden cost of COVID-19: focus on antimicrobial resistance in bloodstream infections. Microorganisms. 11:1299. 10.3390/microorganisms11051299 37317274 PMC10222833

[B17] Polemis M. Mandilara G. Pappa O. Argyropoulou A. Perivolioti E. Koudoumnakis N.. (2021). COVID-19 and antimicrobial resistance: data from the Greek electronic system for the surveillance of antimicrobial resistance—WHONET-Greece (January 2018–March 2021). Life 11:996. 10.3390/life11100996 34685368 PMC8538738

[B18] Ra Y. E. Bang Y. J. (2024). Balancing act of the intestinal antimicrobial proteins on gut microbiota and health. J. Microbiol. 62, 167–179. 10.1007/s12275-024-00122-3 38630349 PMC11090926

[B19] Reffat N. Schwei R. J. Griffin M. Pop-Vicas A. Schulz L. T. Pulia M. S. A.. (2024). Scoping review of bacterial resistance among inpatients amidst the COVID-19 pandemic. J Glob Antimicrob Resist. 38, 49–65. 10.1016/j.jgar.2024.05.010 38789083 PMC11392638

[B20] Smail S. W. Albarzinji N. Salih R. H. Taha K. O. Hirmiz S. M. Ismael H. M.. (2025). Microbiome dysbiosis in SARS-CoV-2 infection: implication for pathophysiology and management strategies of COVID-19. Front. Cell Infect Microbiol. 15:1537456. 10.3389/fcimb.2025.1537456 40330025 PMC12052750

[B21] Tacconelli E. Carrara E. Savoldi A. Harbarth S. Mendelson M. Monnet D. L.. (2018). Discovery, research, and development of new antibiotics: the WHO priority list of antibiotic-resistant bacteria and tuberculosis. Lancet Infect Dis. 18, 318–327. 10.1016/S1473-3099(17)30753-3 29276051

[B22] Toc D. A. Botan A. Botescu A. M. C. Brata V. D. Colosi I. A. Costache C.. (2023). Tale of two pandemics: antimicrobial resistance patterns of *Enterococcus* spp. in COVID-19. Antibiotics 12:312. 10.3390/antibiotics12020312 36830223 PMC9952321

[B23] Toc D. A. Mihaila R. M. Botan A. Bobohalma C. N. Risteiu G. A. Simut-Cacuci B. N.. (2022). Enterococcus and COVID-19: The emergence of a perfect storm? Int J Transl Med. 2, 220–229. 10.3390/ijtm2020020

[B24] Wang M. Zhang Y. Li C. Chang W. Zhang L. (2023). The relationship between gut microbiota and COVID-19 progression: new insights into immunopathogenesis and treatment. Front Immunol. 14:1180336. 10.3389/fimmu.2023.1180336 37205106 PMC10185909

[B25] Weiner-Lastinger L. M. Pattabiraman V. Konnor R. Y. Patel P. R. Wong E. Xu S. Y.. (2022). The impact of coronavirus disease 2019 (COVID-19) on healthcare-associated infections in 2020: a summary of data reported to the National Healthcare Safety Network. Infect Control Hosp. Epidemiol. 43, 12–25. 10.1017/ice.2021.362 34473013

[B26] Zhang F. Lau R. I. Liu Q. Su Q. Chan F. K. L. Ng S. C.. (2023b). Gut microbiota in COVID-19: key microbial changes, potential mechanisms and clinical applications. Nat. Rev. Gastroenterol. Hepatol. 20, 323–337. 10.1038/s41575-022-00698-4 36271144 PMC9589856

[B27] Zhang X. Tan L. Ouyang P. Ma H. Peng J. Shi T.. (2023a). Analysis of distribution and antibiotic resistance of Gram-positive bacteria isolated from a tertiary-care hospital in southern China: an 8-year retrospective study. Front Microbiol. 14:1220363. 10.3389/fmicb.2023.1220363 37840716 PMC10568454

